# The Effects of Dietary Fiber and High-Dietary-Fiber Diets on Body Weight Regulation and Lipid Metabolism in Athletes: A Narrative Review

**DOI:** 10.3390/nu18172832

**Published:** 2026-08-28

**Authors:** Fantao Fang, Tingting Hou, Yonglin Chen, Manjiang Hu, Boling Gu, Zhenwei Li, Zhuixin Wang, Yu-Heng Mao

**Affiliations:** 1 School of Physical Education, Guangzhou Sport University, Guangzhou 510500, China; dandanfft@163.com; 2 School of Exercise and Health, Guangzhou Sport University, Guangzhou 510500, China; 17852776927@163.com (T.H.); chenyl@163.com (Y.C.); 11474@gzsport.edu.cn (M.H.); 202306060124@stu.gzsport.edu.cn (B.G.); 202306060125@stu.gzsport.edu.cn (Z.L.); 3 Guangdong Provincial Key Laboratory of Human Sports Performance Science, Guangzhou Sport University, Guangzhou 510500, China

**Keywords:** dietary fiber, athletes, weight regulation, high-dietary-fiber diets, gut microbiota, lipid metabolism, gastrointestinal symptoms, adipose tissue

## Abstract

The beneficial effects of dietary fiber (DF) and high-dietary-fiber diets (high-DF diets) on body weight (BW) regulation and lipid metabolism have been extensively investigated in the general population. However, due to the unique metabolic adaptations of athletes, exercise-specific nutritional requirements, and enhanced gastrointestinal sensitivity caused by intensive training and competition, the evidence for their application and in-depth mechanism remains limited. This review synthesizes current evidence on the effects of DF and high-DF diets on BW regulation and lipid metabolism in athletic populations. Emerging data indicate that athletes exhibit unique lipid metabolic phenotypes and gut microbial compositions relative to non-athletic individuals—factors that may modulate their physiological responses to DF interventions. Studies conducted specifically in athletes suggest that DF supplementation and high-DF diets—including the vegetarian diet and the Mediterranean diet—may support favorable changes in body composition and lipid-related biomarkers. Nevertheless, these benefits appear to be moderated by sport discipline, biological sex, and interindividual variability in metabolic and gastrointestinal physiology. Critically, excessive or poorly timed DF intake—particularly during intense training blocks or immediately before or during competition—may exacerbate gastrointestinal discomfort and impair energy availability, thereby potentially compromising performance. Consequently, the integration of DF into athletic nutrition strategies necessitates individualized consideration of fiber type, dosage, timing of ingestion, fermentability, and gastrointestinal tolerance. Future research should prioritize rigorously designed longitudinal studies and randomized controlled trials employing standardized DF interventions and outcome assessments. Comprehensive evaluation—including metabolic parameters, markers of energy availability, gastrointestinal symptomatology, and microbiome profiling—will be essential to inform evidence-based, personalized DF recommendations aligned with athletes’ training phases and metabolic profiles.

## 1. Introduction

Dietary fiber (DF) consists of complex carbohydrate polymers that resist enzymatic digestion and absorption in the human small intestine. Although DF is not hydrolyzed by endogenous digestive enzymes [1], this resistance to digestion is precisely what confers its pivotal physiological roles in body weight (BW) regulation and lipid metabolism [2]. As a primary energy source for the gut microbiota (GM), DF undergoes microbial fermentation in the colon to generate bioactive metabolites—most notably short-chain fatty acids (SCFAs)—which actively modulate lipid metabolism [3]. For example, SCFAs have been demonstrated to activate AMP-activated protein kinase (AMPK), a central cellular energy sensor, thereby suppressing adipocyte lipogenesis and promoting fatty acid oxidation [4]. Moreover, DF enhances satiety through multiple mechanisms, including increased gastric distension and delayed gastric emptying, leading to reduced total energy intake and supporting long-term BW regulation [5]. Consequently, DF has been widely integrated into evidence-based BW regulation strategies and incorporated into a broad range of functional foods and dietary supplements [6,7]. Nevertheless, athletes display distinct patterns of energy expenditure, energy availability, body-composition goals, and training-induced physiological adaptations relative to the general population [8,9,10,11,12]. Moreover, high-intensity exercise triggers splanchnic hypoperfusion and mechanical stress on the gastrointestinal tract, thereby diminishing intestinal tolerance to DF and frequently eliciting gastrointestinal symptoms that compromise athletic performance [2]. Consequently, findings derived from studies in the general population cannot be directly generalized to athletes. However, emerging evidence suggests that athletes may derive comparable benefits from DF supplementation or high-DF diets—including improvements in BW regulation and lipid metabolism [13,14]. Furthermore, modulation of the gut microbiota (GM) through DF intake has been proposed to support fatigue recovery and potentially augment athletic performance [13]. However, in this population, the substantial gastrointestinal stress induced by high-intensity training and competition—compounded by the intrinsic physiological properties of DF, such as gas production during colonic fermentation and osmotic water retention—commonly precipitates exercise-related gastrointestinal symptoms, including abdominal bloating, cramping, and general discomfort, which may ultimately compromise athletic performance [15]. Accordingly, this review seeks not only to establish a theoretical foundation for future research on the effects of DF and high-DF diets on BW regulation and lipid metabolism in athletes, but also to inform the development of evidence-based, practical dietary strategies that mitigate gastrointestinal distress while preserving the beneficial modulatory effects of DF on the GM.

## 2. Methods

This narrative review was synthesized from a systematic literature search conducted in January 2026 across PubMed, Web of Science, and Google Scholar, covering publications from January 2000 to July 2026. The search strategy combined MeSH terms and free-text keywords: (“dietary fiber” OR “fiber” OR “prebiotic” OR “inulin” OR “resistant starch”) AND (“athlete” OR “sports” OR “exercise” OR “physical activity” OR “active individual”) AND (“weight management” OR “weight control” OR “body composition” OR “fat mass” OR “obesity”) AND (“gut microbiota” OR “lipid metabolism” OR “appetite” OR “inflammation”). We included peer-reviewed studies (English or Chinese) involving athletes or healthy physically active individuals, investigating high-fiber dietary patterns or interventions on body weight, body composition, lipid metabolism, appetite, or gut microbiota. We excluded non-peer-reviewed articles, studies on severe metabolic disorders (e.g., diabetes, cancer), and inaccessible full texts. Given the narrative nature of this review and the heterogeneity of the included evidence, we did not employ a formal structured screening protocol. Instead, we prioritized findings from higher-quality study designs (e.g., systematic reviews and randomized controlled trials) in our interpretation, while mechanistic and animal studies were used to support biological plausibility.

## 3. Differences in Energy Metabolism Between Athletes and Non-Athletes—Lipid Metabolism and Gut Microbiota

In BW regulation, lipid metabolism—encompassing processes related to fat and lipid homeostasis—is a key modulating factor. However, it is critical to emphasize that lipid metabolism does not operate in isolation; rather, it engages in intricate, bidirectional crosstalk with multiple physiological systems, with the gut system playing a particularly central role [16,17,18]. Moreover, chronic exercise training elicits robust metabolic adaptations that reshape lipid storage, mobilization, and oxidative utilization—potentially resulting in divergent physiological responses to dietary interventions targeting energy balance and lipid metabolism in athletes compared with their non-athletic counterparts.

### 3.1. Differences in Lipid Metabolism

**Figure 1:**
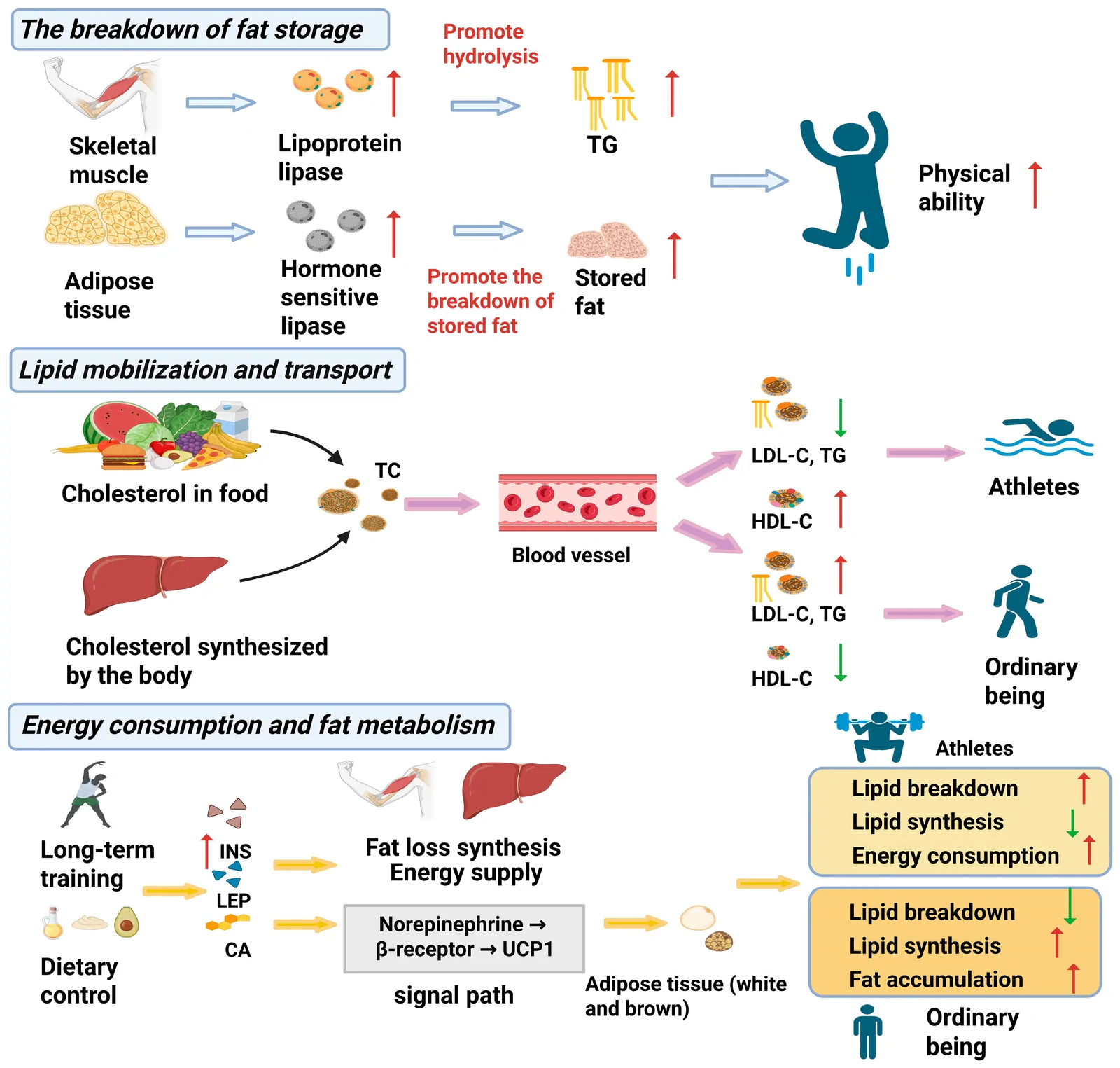
A diagram illustrating the impact of differences in lipid metabolism among athletes. Lipid metabolism regulates an athlete’s body composition. Exercise and diet influence weight and body fat changes by reshaping this process. TG, triglycerides; TC, total cholesterol; LDL-C, low-density lipoprotein cholesterol; HDL-C, high-density lipoprotein cholesterol; LEP, leptin; INS, insulin; CA, catecholamine; UCP1, uncoupling protein 1. Red arrow: indicates an increase in the index; green arrow: indicates a decrease in the index.

Lipid metabolism encompasses the synthesis, transport, storage, and catabolism of lipids—including fatty acids, triglycerides (TG), and cholesterol—within the human body. These processes play a critical role in regulating BW and body-fat composition in athletes (Figure 1). However, the physiological adaptations associated with enhanced lipid metabolism extend far beyond superficial anthropometric measures such as BW and body-fat percentage (BF%). In athletes, circulating lipid concentrations—as well as the hormonal sensitivity of skeletal muscle and adipose tissue—undergo dynamic, coordinated adjustments in response to acute and chronic metabolic demands [8,9,19].

With regard to lipid transport and distribution in the blood, long-term regular exercise induces a cardioprotective blood-lipid profile in athletes. Compared with age-matched non-athletes, athletes consistently exhibit lower circulating concentrations of TG, low-density lipoprotein cholesterol (LDL-C), and total cholesterol (TC), whereas their high-density lipoprotein cholesterol (HDL-C) levels are either comparable to or elevated relative to those of sedentary peers [20]. In contrast, non-athletes—due to insufficient physical activity—are more prone to a chronic imbalance between macronutrient intake and energy expenditure. When dietary intake of fat, particularly saturated fatty acids (SFAs), exceeds the metabolic demands imposed by physical activity, circulating levels of TC, TG, and LDL-C may increase substantially, thereby elevating the risk of obesity and cardiovascular disease [10,11].

With regard to the regulation of key enzymes and hormones involved in lipid metabolism, lipoprotein lipase (LPL) activity is significantly elevated in the skeletal muscle of athletes [21]. This enhanced LPL activity facilitates the efficient hydrolysis of TG carried by very-low-density lipoprotein (VLDL), thereby increasing fatty acid availability for muscular energy production [22]. Concurrently, heightened hormone-sensitive lipase (HSL) activity in adipose tissue augments lipolysis and promotes the mobilization of free fatty acids [23]. Regarding hormonal regulation, athletes exhibit markedly improved insulin sensitivity [8,9,24], which—by suppressing hepatic gluconeogenesis—indirectly attenuates de novo lipogenesis. In addition, compared with a control group of non-athletes of the same age, athletes have relatively lower circulating leptin levels [12,25].

With regard to adipose tissue distribution and function, athletes exhibit not only a reduced capacity for fat storage but also enhanced thermogenic activity. This phenotype arises from heightened activation of brown adipose tissue (BAT) and upregulated expression of uncoupling protein 1 (UCP1), collectively increasing resting energy expenditure in response to training-induced sympathetic nervous system stimulation [26,27,28,29].

Consequently, long-term exercise training fosters an intrinsic regulatory network characterized by enhanced lipid oxidative capacity and metabolic flexibility. Nevertheless, although these inherent physiological adaptations establish a favorable baseline for energy homeostasis, athletes frequently require further optimization of body composition or augmentation of fat oxidation to meet dynamic energy demands across training and competition contexts. This underscores the need to explore complementary metabolic pathways that extend beyond conventional training-induced adaptations. In this context, emerging evidence highlights the GM as a critical, diet-responsive modulator of systemic lipid metabolism in athletes [18,30,31]. Specifically, gut-derived metabolites—particularly SCFAs—have been shown to regulate hepatic lipogenesis and promote peripheral fatty acid oxidation through AMPK-dependent signaling pathways [3,32]. Accordingly, the subsequent sections will focus on DF as the primary fermentable substrate underpinning these gut–lipid interactions, thereby presenting a promising adjunctive strategy for enhancing metabolic efficiency and optimizing body composition in athletic populations.

### 3.2. Differences in Gut Microbiota

Compared with non-athletic populations, athletes have been reported to exhibit distinct gut microbial characteristics [30,33,34]. For example, athletes may display greater gut microbial diversity, together with alterations in the abundance of specific microbial taxa (i.e., key or dominant biological groups at the genus or family level). These microbial features are linked to alterations in the production of specific microbiota-derived metabolites and their associated metabolic pathways, suggesting that the GM may contribute to the metabolic adaptations observed in athletes. Such microbial characteristics likely arise from the synergistic effects of physiological adaptations induced by long-term exercise training and habitual dietary patterns—collectively shaping a gut microbial ecosystem aligned with the metabolic demands of physical activity.

First, elite athletes demonstrate significantly greater gut microbial diversity. Previous studies have identified 22 bacterial phyla, 68 families, and 113 genera in the GM of rugby players—substantially more than the 11 phyla, 33 families, and 65 genera detected in non-athletic control populations [30]. Higher microbial α-diversity may be associated with more complex and resilient microbial ecosystems, which may lead to greater ecological stability and a broader range of metabolic capabilities. Second, athlete-associated shifts in specific microbial taxa have been consistently observed, particularly among species implicated in intestinal barrier integrity, SCFA production, and host metabolic health—including *Akkermansia*, *Roseburia*, and *Faecalibacterium prausnitzii* [33]. These taxa are well-documented for their roles in producing microbiota-derived metabolites and modulating host metabolic regulation. For instance, competitive cyclists exhibit an elevated relative abundance of *Prevotella*, which correlates with the enrichment of microbial metabolic pathways involved in amino acid and carbohydrate metabolism [34]. Moreover, compared with amateur cyclists, competitive cyclists display a significantly higher abundance of *Methanobrevibacter smithii*, concomitant with distinct alterations in methane metabolism and carbohydrate metabolism-related pathways [34].

Collectively, these findings suggest that the gut microbial features observed in athletes may be linked to functions related to energy metabolism. However, it is important to note that elevated microbial diversity or the enrichment of specific taxonomic groups does not inherently reflect a superior metabolic phenotype. The physiological relevance of these microbial characteristics remains contingent upon multiple interrelated factors—including sport discipline, training volume and intensity, dietary habits, and host-specific attributes.

### 3.3. Regulation of Lipid Metabolism by Gut Microbiota

The role of the GM in regulating lipid metabolism is now well established in fundamental research. Early studies utilizing germ-free, conventionally raised, and high-fat-diet-fed mouse models confirmed this regulatory influence by comparing fatty acid oxidation efficiency—as well as circulating concentrations of TG, high-density lipoprotein (HDL), cholesterol, and total cholesterol—across experimental groups [35,36,37]. Functioning as a “hidden metabolic organ,” the GM synthesizes a diverse array of bioactive metabolites. Upon entering the systemic circulation, these metabolites trigger cascades of metabolic signaling pathways and thereby actively modulate both lipid synthesis and catabolism in a manner functionally analogous to that of classical hormones [32,38].

Notably, several studies have reported associations between gut microbial features and parameters related to lipid metabolism in athletes (Table 1). For instance, elite male rugby players exhibit increased abundances of the bacterial genera *Akkermansia* and *Prevotella*, concomitant with elevated plasma levels of L-carnitine—an amino acid-derived metabolite that facilitates the mitochondrial import of fatty acids for oxidative metabolism—as well as enhanced capacity for fatty acid β-oxidation [30,39]. Moreover, shifts in the relative abundances of these taxa correlate significantly with lower BF% and higher lean body mass (LBM), suggesting that athlete-associated gut microbial signatures may contribute to favorable body composition and metabolic phenotypes [30,31].

Similarly, a study reported that professional athletes exhibited significantly higher abundances of several bacterial taxa—including *Prevotella*, *Alistipes*, *Bacteroides*, and *Bifidobacterium*—compared with both esports athletes and general physical education students [40]. Concurrently, microbial functional pathways linked to fatty acid biosynthesis, fatty acid degradation, and the tricarboxylic acid (TCA) cycle were significantly enriched. Furthermore, elevated abundance of *Parabacteroides* has been observed in elite Wushu athletes and was associated with shifts in functional pathways involved in complex lipid metabolism and bile acid metabolism [41]. Mechanistic studies have demonstrated that bile acids, acting as endogenous ligands for the farnesoid X receptor (FXR), regulate hepatic lipid synthesis and VLDL-associated triglyceride secretion, thereby contributing to systemic lipid homeostasis [42,43]. Nevertheless, it is important to emphasize that current evidence from athlete-based studies primarily reveals correlative associations between gut microbial composition and metabolic pathways; whether specific microbial taxa directly enhance lipid metabolism in athletes remains to be experimentally validated.

In summary, the gut microbial ecosystem of athletes is closely linked to lipid metabolism. As a key dietary component, DF plays a pivotal role in shaping gut microbial composition and modulating microbiota-derived metabolites [44]. Consequently, DF-based dietary interventions aimed at modulating lipid metabolism may represent a promising strategy for BW regulation in athletes. However, inter-individual variability—arising from long-term exercise training and habitual dietary patterns—may lead to heterogeneous responses in lipid metabolism and BW regulation following identical high-DF dietary interventions among athletes.

**Table 1:** Gut microbiota and lipid metabolism of athletes.

Athlete	Control Group	Gut Bacteria (Key Taxa)	Metabolism (Metabolites)	Lipid Metabolism	Ref.
Rugby players (*N* = 40; all male)	High BMI healthy individuals (*N* = 24, all male); Low BMI healthy individuals (*N* = 22, all male)	1. Athletes compared to the overall control group: Increased abundance of *Prevotellaceae* and *S24-7*; 2. Athletes compared to the high BMI control group: Increased abundance of *Succinivibrionaceae*, *Akkermansiaceae* and *Erysipelotrichaceae*; 3. Athletes compared to low BMI control group: Reduced abundance of *Lactobacillaceae*; 4. High BMI compared to low BMI: Increased abundance of *Dorea* and *Pseudobutyrivibrio*; Decreased abundance of *Ruminococcaceae Incertae Sedis* and *Akkermansia.*	Compared to the two control groups: Athletes exhibited increased levels of acetate, propionate, butyrate, and valerate, alongside reduced levels of succinate.	The lipid biosynthesis pathway in the high BMI control group was higher than that in the athlete group.	[30,31]
Professional athletes (*N* = 25; all male; comprising 8 marathon runners, 3 cross-country skiers, and 14 footballers)	Sports science students (*N* = 36, all male) Esports players (*N* = 109, all male)	1. Athletes compared to esports players and students: Increased abundance of *Akkermansia*, *Roseburia hominis*, *Anaeromassilibacillus* sp. An250, and the *Veillonella* genus; Decreased abundance of *Bilophila wadsworthia* and *Ruminococcus gnavus*; 2. Sports science students compared to esports players: Increased abundance of *Parabacteroides distasonis* and *Lachnospira pectinoschiza*.	1. Athletes compared to esports players and sports science students: Differences in 77 and 158 metabolic pathways (involving fermentation, amino acid synthesis and degradation, carbohydrate synthesis and degradation, fatty acid synthesis and degradation, and the tricarboxylic acid cycle); 2. Athletes and sports science students compared to esports players: Methionine distinguishes esports players from the other two groups.	1. Athletes compared to esports players and sports science students: exhibit richer fatty acid synthesis and degradation pathways; 2. Esports players compared to sports science students: consume higher levels of saturated FA and lower levels of polyunsaturated FA.	[40]
Endurance athletes (*N* = 14; all male; 14 elite marathon runners, 11 cross-country skiers)	General population (*N* = 45; 14 males, 31 females)	1. Compared to the control group of non-athletes, athletes exhibited reduced abundance of *Bacteroidetes*, increased abundance of *Prevotella*, and an elevated *Firmicutes*/*Bacteroidetes* (F/B) ratio; 2. Skiers demonstrated the highest GM biodiversity; 3. Marathon runners showed over fourfold increased abundance of *Veillonella* compared to the control group.	1. Common differential pathways: Enhanced primary bile acid biosynthesis capacity and improved D-alanine metabolism; 2. Marathon runners’ unique differential pathways: Enhanced galactose metabolism and arachidonic acid metabolism capacity.	Marathon runners and skiers exhibit significantly different bile acid synthesis capabilities compared to a control group of ordinary individuals.	[42]
Professional cyclists (*N* = 22; 14 males; 8 females)	Amateur cyclists (*N* = 11; 8 males, 3 females)	1. *Methanobrevibacter smithii* increased in the occupational group compared with the non-occupational group; 2. *Prevotella* abundance greater than or equal to 2.5% was positively associated with exercise duration greater than 11 h per week.	Increased levels of propionic acid, butyric acid, and secondary bile acids.	Primary bile acid biosynthesis pathways were enhanced.	[34]

BMI, Body mass index; FA, fatty acid; F/B, Firmicutes/Bacteroidetes.

## 4. Classification and Functions of Dietary Fiber

DF comprises a diverse class of complex carbohydrate polymers that resist hydrolysis by endogenous human digestive enzymes—such as α-amylase—encoded in the human genome. It modulates host health through multiple physicochemical properties, including solubility, viscosity, and fermentability (Table 2) [45].

On the basis of water solubility, DF is conventionally classified into soluble dietary fiber (SDF) and insoluble dietary fiber (IDF). SDF forms a viscous solution that delays gastric emptying and attenuates nutrient absorption through both physical mechanisms—such as the formation of gel networks—and chemical interactions—including water retention and swelling [46,47]. Its porous architecture and abundant hydrophilic functional groups enhance hydration capacity, inhibit α-glucosidase activity, and contribute to the regulation of blood glucose and lipid metabolism, partly via adsorption of bile acids [48,49,50]. IDF—exemplified by wheat bran and goji berry (*Lycium barbarum*) pulp fiber—exerts its primary physiological effects through water-holding capacity and swelling: upon hydration, IDF typically increases in volume two- to three-fold, thereby increasing fecal bulk and promoting intestinal peristalsis [51,52]. Concurrently, IDF binds cholesterol and other potentially harmful compounds—including endotoxins and heavy metals—via hydrogen bonding and van der Waals interactions; these complexes are subsequently excreted in the feces [53,54]. Furthermore, fermentable fibers—particularly SDF—are metabolized by the GM to produce SCFAs, which modulate the intestinal microenvironment and support systemic metabolic homeostasis [55,56]. Nevertheless, it is important to emphasize that DF within the same solubility category may elicit distinct and multifaceted metabolic responses, attributable to inter-fiber differences in solubility, viscosity, fermentability, and dose-dependent effects [44]. Moreover, the application of DF in athletic nutrition must account for both the food matrix in which the DF is delivered and inter-individual variations in GM composition—factors that collectively shape physiological and metabolic responses to DF intake [44,57].

Despite these multifaceted benefits, the high intensity and frequency of training and competition often lead athletes to restrict their intake of DF sources that are poorly digestible or poorly absorbed—thereby minimizing the risk of gastrointestinal symptoms, including abdominal pain, bloating, and discomfort [57]. In practice, however, professional sports nutritionists routinely modulate the DF content of athletes’ diets to align with short-term or individualized BW goals, thereby supporting optimal on-field performance [58].

**Table 2:** Physical and chemical properties of common dietary fibers.

Fiber Category	Fiber Type	Physical and Chemical Properties			General Chemical Structures	Ref.
		Solubility	Viscosity	Fermentability		
Resistant dextrins	Polydextrose	Soluble	Low	Slow to moderate	α-1,6, β-1,6	[59]
	Resistant maltodextrin	Soluble	Low	Slow to moderate	α-1,2, α-1,3	[60,61]
Resistant starch	RS-1	Insoluble	Non-viscous	High	α-1,4, α-1,6	[62]
	RS-2	Soluble	Non-viscous	High	α-1,4, α-1,6	[62]
	RS-3	Soluble	Non-viscous	High	α-1,4, α-1,6	[63]
	RS-4	Soluble	Low to medium	High	α-1,4, α-1,6	[64]
	RS-5	Soluble	Low	Low	α-1,4, α-1,6	[65]
Oligosaccharides	Galactooligosaccharides	Soluble	Low	High	β-1,4, β-1,6	[66]
	Fructooligosaccharides	Soluble	Low	High	β-2,1	[67]
	Xylooligosaccharides	Soluble	Low	Moderate to high	β-1,4	[68]
	Arabinoxylooligosaccharides	Soluble	Low	Moderate to high	β-1,4, α-1,2, α-1,3	[69]
Non-starch polysaccharides	Arabinoxylans	Soluble	Medium	High	β-1,4, α-1,2, α-1,3	[70]
	β-Glucans	Soluble	Moderate to high	Moderate Slow to moderate	β-1,3	[71,72]
	Inulin	Soluble	Low to high	High	β-2,1	[73]
	Cellulose	Insoluble	Non-viscous	Poor	β-1,4	[74]
Other fibers	Lignin	Insoluble	Non-viscous	Poor	β-O-4	[75]
	Alginate	Soluble	High	Poor to slow	β-1,4	[76]
	Arabinogalactan	Soluble	Low to moderate	Slow to moderate	β-1,3, β-1,6	[77]
	Galactomannan	Soluble	Moderate to high	Slow to moderate	β-1,4	[78]
	Glucomannan	Soluble	High	Slow to moderate	β-1,4	[79]

RS-1: resistant starch type 1; RS-2, resistant starch type 2; RS-3, resistant starch type 3; RS-4, resistant starch type 4; RS-5, resistant starch type 5.

## 5. Dietary Fiber and High-DF Diets Regulate Body Weight and Lipid Metabolism in Athletes

As a class of indigestible carbohydrates in humans, DF has long attracted extensive research interest due to its pivotal role in BW regulation [2,80,81,82,83]. However, elite athletes face more complex BW management goals that require concurrent optimization of body composition. Specifically, beyond reducing BF%, athletes must preserve—and often increase—LBM to enhance performance, while simultaneously maintaining adequate energy availability and recovery capacity to sustain high-intensity training. In this context, the physiological role of DF becomes especially critical.

### 5.1. Dietary Fiber and High-DF Diets in Athletes

Currently, several studies have confirmed the role of DF and high-DF diets in regulating body weight (BW) and promoting lipid metabolism in athletes (see Table 3), although the physiological effects of DF—including BW regulation, reduction in fat mass (FM), and improvement of blood lipid profiles—have been well established in non-athletic populations [84]. However, evidence in athletic populations remains relatively limited, and observed benefits have not consistently mirrored those reported in non-athletes. For instance, an eight-week DF intervention in basketball players demonstrated reductions in both BW and BF% in the high-dose (6.84 g/day) and low-dose (3.24 g/day) groups [13]. Similarly, a four-week supplementation study with β-glucan (2 g/day) in table tennis players reported an increase in upper-limb lean mass [85]. Notably, however, no significant improvements were observed in conventional blood lipid markers—including TC, TG, and LDL-C—in either study. This lack of change may reflect the already favorable baseline lipid profiles typical of healthy, physically active individuals, thereby limiting the scope for further clinically meaningful reductions. In this population, the primary benefit of DF intake appears to lie not in dramatic metabolic shifts, but rather in the maintenance of BW and lipid homeostasis—while concurrently preserving or even enhancing exercise capacity and athletic performance. For example, in the aforementioned study of table tennis players, β-glucan supplementation not only modulated BW and lipid metabolism but also significantly activated the creatine metabolic pathway, increased maximal oxygen uptake (VO_2_ max), and suppressed tumor necrosis factor-alpha (TNF-α)—a key pro-inflammatory cytokine—ultimately resulting in improved handgrip strength [85].

Compared with isolated DF supplementation, a high-DF diet offers athletes not only a greater total DF intake but also the synergistic fermentation effects conferred by diverse DF types and the complementary physiological actions of co-occurring micronutrients. A high-DF diet is defined as a dietary pattern characterized by daily DF intake substantially exceeding the general population’s recommended intake (>25–35 g/day) and derived predominantly from whole plant foods—including whole grains, legumes, vegetables, fruits, and nuts [86]. Vegetarian diets (including vegan diets) and the Mediterranean diet represent prominent exemplars of this dietary pattern.

The vegetarian diet (VD) is a plant-based dietary pattern in which energy is predominantly derived from seeds, fruits, other edible plant tissues, and plant-derived foods [87]. For instance, an 8-week VD intervention—characterized by a daily DF intake exceeding 36.7 g—in football players demonstrated that, relative to an omnivorous control group, the vegetarian group exhibited significant reductions in BW and BF%, along with lower serum concentrations of TG, TC, and LDL-C [14]. The Mediterranean diet (MD), in turn, is a well-established dietary pattern characterized by high intakes of fruits, vegetables, whole grains, legumes, and nuts, and is widely recognized for its nutritional balance and rich DF content [88]. For example, a 6-week MD intervention conducted in retired athletes resulted in a significant reduction in BW and a concomitant increase in the Omega-3 Index [89]. The Omega-3 Index is defined as the combined percentage of eicosapentaenoic acid (EPA) and docosahexaenoic acid (DHA)—two biologically critical Omega-3 fatty acids—relative to total fatty acids in erythrocyte membranes [90]. As integral structural components of mitochondrial membrane phospholipids, DHA and EPA enhance mitochondrial uptake of long-chain fatty acids by increasing membrane fluidity and upregulating the activity of carnitine palmitoyltransferase 1 (CPT-1), the rate-limiting enzyme responsible for facilitating the translocation of long-chain fatty acids into the mitochondria for β-oxidation [91,92].

In addition, high-DF diets—defined solely by an increased DF content—have also demonstrated beneficial effects on BW and lipid metabolism in athletes [93,94]. For instance, a study incorporating legumes into the diet (providing 41 ± 17 g/day of DF) reported that female football players experienced a reduction in FM without concomitant changes in BW, alongside significant improvements in HDL-C (legume-based diet: +0.5 ± 0.7 mmol/L; regular diet: −0.6 ± 0.3 mmol/L) and in the TC-to-HDL-C ratio (legume-based diet: −2.4 ± 2.9; regular diet: +2.6 ± 2.2) [93]. However, these favorable changes in the blood lipid profile were not observed in male football players. This sex-specific response may be attributable, at least in part, to estrogen. Evidence suggests that individuals with higher circulating estrogen concentrations exhibit greater sensitivity to dietary interventions aimed at improving lipid profiles [95]. A similar sex-dimorphic effect has been documented in non-athletic populations. In a randomized controlled trial supplementing the diet with inulin and resistant maltodextrin (10 g/day), women derived significantly greater benefits from the DF intervention than men [96]. Specifically, women showed greater improvements in hip circumference, the hepatic injury marker alanine aminotransferase (ALT), and secondary bile acid concentrations. In contrast, men exhibited improvements only in hepatic fat content and insulin sensitivity—neither of which translated into measurable changes in body composition. Collectively, these findings suggest that the metabolic benefits of DF supplementation on BW regulation and lipid metabolism in athletes are sex-dependent, with certain cardiometabolic and hepatic markers demonstrating more pronounced improvements in women.

**Table 3:** The effects of dietary fiber and high-dietary-fiber diets on body weight regulation and lipid metabolism in athletes.

Athletic Event	Research Type	Sample Size	Gender	Type of DF/DF Diet	DF Intake	Duration	Main Manifestations	Ref.
Gaelic football	Case trial	1	Male	Vegan diet	68 g/d	About 8 months	LBM increased by 3.64 kg; BF% decreased from 11.3% to 10.4%.	[97]
Marathon, triathlon, cling	Cross-sectional trial	70	40 males, 30 females	Vegetarian diet, omnivore diet	38 ± 13 g/d, 24 ± 9 g/d	≥3 months	BW and LBM significantly lower in vegetarians.	[98]
Football	Randomized crossover trial	19	7 males, 12 females	Pulse-based Diet Regular diet	41 ± 17 g/day, 24 ± 9 g/day	4 weeks	Increased levels of HDL and a decreased ratio of TC to HDL were found only in females.	[93]
Table tennis	Randomized controlled trial	29	/	β-glucan	2 g/day	4 weeks	Upper-LBM showed a slight increase.	[85]
Rhythmic gymnastics, football, middle- and long-distance running, figure skating	Single-arm trial	29	All females	Inulin and lactulose	2.5 g/day and 1 g/day	12 weeks	Improved bone health while maintaining BW.	[99]
Football	Non-randomized controlled trial	18	All males	Vegan diet	>36.7 g/d	8 weeks	Compared to the omnivorous group, the vegan group showed reductions in BW and BF%, as well as lower levels of TG, TC, and LDL-C in the blood.	[14]
Leisure endurance running, resistance training	Randomized crossover trial	22	12 males, 10 females	WFPB diet, PBMA diet, omnivorous diet	40 ± 12 g/d, 31 ± 10 g/d, 25 ± 11 g/d	Each diet lasted for 4 weeks.	Compared with the omnivorous diet, runners and resistance trainers on the WFPB diet showed significant decreases in both BW and BF%; on the PBMA diet, only runners showed a significant decrease in BF%.	[94]
Basketball	Randomized controlled trial	20	All males	High-dose DF supplement, Low-dose DF supplement	23.1 ± 7.8 g/d, 19.5 ± 7.8 g/d	8 weeks	Improvements in BW, BF%, and FM were more pronounced in the high-dose group.	[13]
Volleyball	Single-arm trial	11	All males	Mediterranean diet	/	About 8 months	Compared with baseline, FFM, SMM, BCMI, TBW, BMR, FM, BMI, and phase angle all increased significantly.	[100]
Leisure endurance running, resistance training	Randomized crossover trial	36	20 males, 15 females, 1 non-binary	Plant-based diet, omnivore diet	28.6 ± 9.74 g/d, 18.1 ± 7.45 g/d	Each diet lasted for 4 weeks.	Among runners, the plant-based diet group experienced a significant reduction in BW compared to the omnivorous group.	[101]
Beach handball	Cross-sectional trial	59	38 males, 21 females	Mediterranean diet	/	/	Female athletes with lower adherence to the Mediterranean diet had higher BW and lower LBM.	[102]
Football (adolescent)	Cross-sectional trial	132	All males	Mediterranean diet	/	/	KIDMED inversely correlated with BMI.	[103]
Handball	Cross-sectional trial	133	All males	Mediterranean diet	/	/	KIDMED score correlated weakly and negatively with BW, BMI, and ∑3-girths sum.	[104]
Retired athletes	Randomized controlled trial	94	All males	Mediterranean diet	/	6 weeks	BMI decreased significantly. In the subgroups with BW loss of 1.5–2.4 kg and >2.4 kg, both FM and WC decreased significantly.	[105]

DF, dietary fiber; LBM, lean body mass; BF%, body fat percentage; BW, body weight; HDL, high-density lipoprotein; TC, total cholesterol; TG, triglycerides; LDL-C, low-density lipoprotein cholesterol; WFPB, whole food plant-based; PBMA, plant-based meat alternatives; FM, fat mass; FFM, fat-free mass; SMM, skeletal muscle mass; BCMI, body cell mass index; TBW, total body water; BMR, basal metabolic rate; KIDMED, Mediterranean diet quality index for children and adolescents; WC, waist circumference.

### 5.2. The Synergistic Effect of Dietary Fiber and Other Nutrients

**Figure 2:**
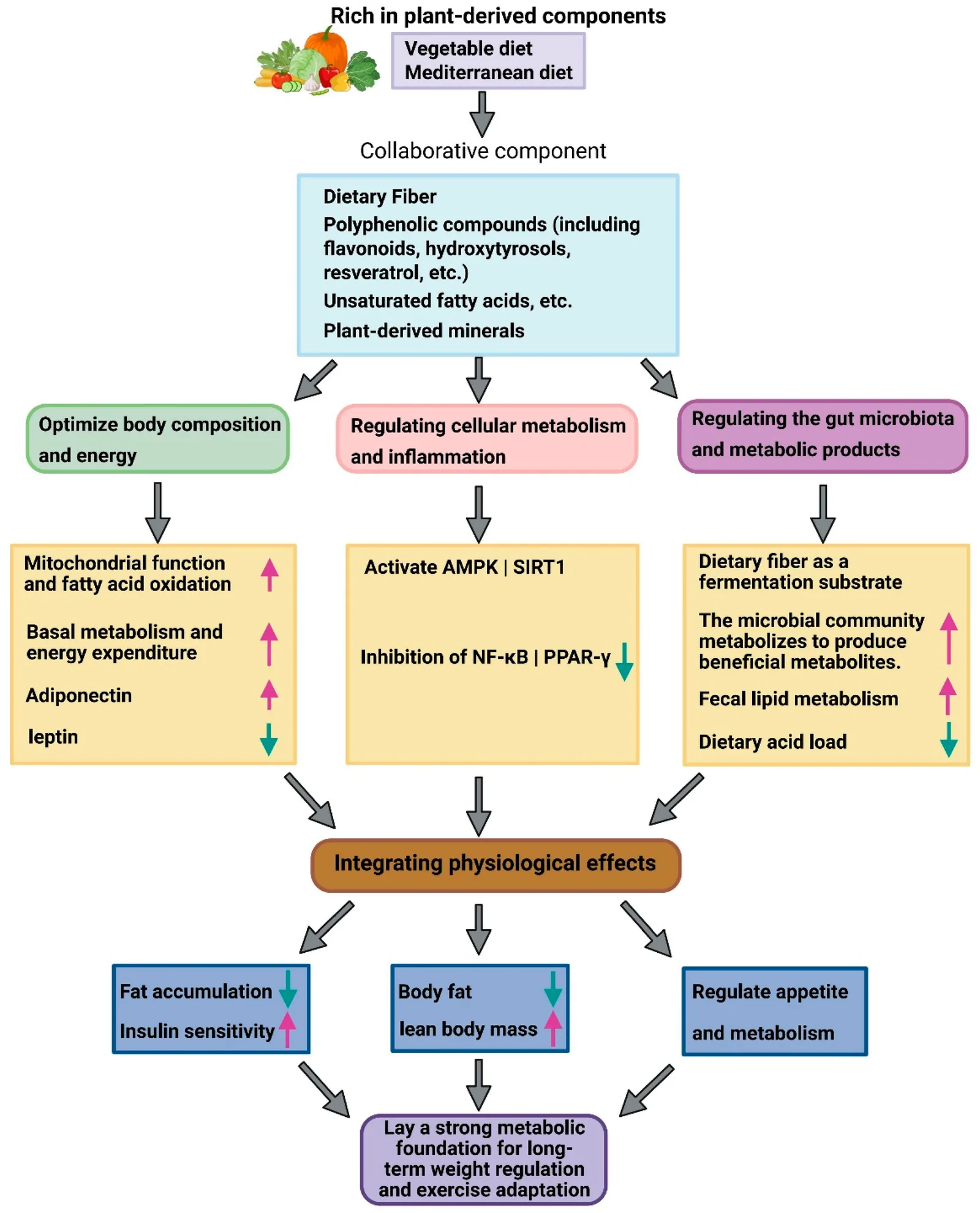
Body weight and lipid metabolism regulation under the influence of a synergistic effect: Multi-factor co-regulation of a high-DF diet; AMPK, AMP-activated protein kinase; SIRT1, sirtuin 1; NF-κB, nuclear factor-kappa B; PPAR-γ, peroxisome proliferator-activated receptor gamma. Red arrow: upward regulatory effect; green arrow: downward regulatory effect.

The improvements in BW regulation and lipid metabolism observed with high-DF diets cannot be attributed solely to DF but rather arise from the synergistic actions of multiple plant-derived bioactive constituents. Accumulating evidence indicates that polyphenolic compounds, minerals, vitamins and carotenoids—abundant in plant-based foods—modulate BW and adipose tissue accumulation through distinct molecular mechanisms (Figure 2) [106,107,108,109,110].

Polyphenolic compounds constitute a class of bioactive phytochemicals that are widely distributed in plants and have garnered substantial scientific interest due to their potential modulatory effects on athletic performance [111,112]. Moreover, accumulating evidence indicates that these compounds play a role in BW regulation and the enhancement of lipid metabolism. For instance, a 10-week randomized supplementation trial employing flavanol-rich cocoa powder (425 mg/day) in athletes reported significant reductions in trunk fat and visceral adipose tissue—changes that were concomitant with a marked increase in circulating follistatin levels [106]. Follistatin is a key endogenous regulatory protein that suppresses the NF-κB-mediated chronic inflammatory pathway and thereby facilitates adipose tissue loss. Mechanistically, this anti-inflammatory action attenuates cytokine-induced impairment of insulin sensitivity and fatty acid oxidation efficiency [113], thus counteracting the inflammation-driven dysregulation of lipid metabolism commonly associated with high-intensity training. Furthermore, follistatin functions as a potent myostatin antagonist, promoting skeletal muscle hypertrophy [114]. Through these complementary actions, follistatin and myostatin inhibition synergistically reduce visceral adipose tissue accumulation, improve systemic lipid profiles, and enhance the relative contribution of fatty acid oxidation to total energy expenditure in athletes.

Minerals act as essential cofactors for numerous metabolic enzymes, thereby enabling DF to participate in glucolipid metabolism, energy homeostasis, and maintenance of electrolyte balance [108]. In a study among female athletes, concurrent supplementation with prebiotic fiber (5 g/day) and ferrous sulfate significantly improved iron status [115]. Moreover, vitamins and carotenoids have been identified as key nutritional factors associated with BW regulation in athletic populations. An eight-week intervention combining vitamin C and quercetin supplementation—though yielding no statistically significant enhancement in exercise performance—was associated with attenuated markers of exercise-induced muscle damage and a reduction in BF% [116]. Separately, a dietary quality assessment conducted in NCAA Division I athletes revealed a moderate inverse correlation between changes in skin carotenoid concentrations and BF% [117].

In fact, high-DF diets also encompass a broad spectrum of other plant-derived bioactive compounds [118,119]. Although direct associations between these constituents and BW regulation or lipid metabolism in athletes remain to be definitively established, accumulating evidence indicates that they exert substantial modulatory effects on both BW and lipid metabolism—likely via alternative, non-fiber-specific mechanistic pathways. For instance, a 16-week randomized intervention with Xanthigen^®^ (a proprietary combination of fucoxanthin-rich brown seaweed extract and pomegranate seed oil; Nektium Pharma S.L., Canary Islands, Spain) in obese women yielded significant reductions in BW, waist circumference, hepatic lipid content, and serum triglyceride concentrations [120]. Mechanistic investigations have demonstrated that Xanthigen attenuates adipose and hepatic lipid accumulation by downregulating peroxisome proliferator-activated receptor-γ (PPAR-γ)—a master transcriptional regulator of lipogenesis [121]—while concurrently increasing resting energy expenditure through the activation of uncoupling protein 1 (UCP-1) in white adipose tissue [121,122]. Collectively, these complementary actions contribute synergistically to the regulation of BW and systemic lipid metabolism.

In addition to the aforementioned micronutrients, the dietary acid load—the systemic acidic or alkaline burden imposed on the body following digestion and fermentation of nutrient components in a high-DF diet—may also modulate BW and lipid metabolism. For example, a study comparing MD and VD used potential renal acid load (PRAL) and net endogenous acid production (NEAP) as quantitative indices of dietary acid load. It reported that changes in PRAL (r = +0.34; *p* = 0.009) and NEAP (r = +0.39; *p* = 0.002) over the first 16 weeks were significantly and positively associated with concurrent changes in BW [123].

In summary, the role of DF in BW regulation extends well beyond its physicochemical properties. As a pivotal ecological modulator of the gut environment, DF acts synergistically with bioactive micronutrient constituents—particularly polyphenols—to influence BW through interconnected metabolic, inflammatory, and GM-mediated pathways. Consequently, for athletes, the strategic adoption of dietary patterns rich in diverse, high-quality DF may support weight loss or long-term weight maintenance.

### 5.3. The Challenge of Dietary Fiber Intake for Athletes

Although current evidence suggests that DF supplementation and high-DF diets may exert beneficial effects on BW regulation and lipid metabolism in athletes, achieving these benefits may require sustained dietary adherence over an extended period. Therefore, the application of DF intake should consider the distinct nutritional demands during competitive and non-competitive phases. During periods of intensive training and competition, excessive DF intake may increase the risk of gastrointestinal symptoms, including bloating, excessive intestinal gas, and abdominal discomfort, which could potentially interfere with training quality and competitive performance. Accordingly, periodic adjustment of DF intake may represent a practical strategy for optimizing both metabolic benefits and gastrointestinal tolerance. For example, Gaelic football players adopted a VD (DF: 68 g/day) and substituted high-DF legumes (e.g., lentils and chickpeas) with low-DF protein sources (e.g., tofu) and low-DF carbohydrate sources (e.g., white rice, pasta, and small potatoes) prior to training and competition [97]. This strategy enabled these athletes to increase LBM throughout the season while maintaining BF%.

In addition to periodically adjusting DF intake, athletes may also consider adopting the dietary principles of a low-FODMAP diet before training or competition. A low-FODMAP diet, which restricts fermentable oligosaccharides, disaccharides, monosaccharides, and polyols, has been widely recognized in gastroenterology as an effective dietary strategy for alleviating gastrointestinal symptoms [124]. A study in endurance athletes demonstrated that, compared with a high-carbohydrate–high-FODMAP diet, a high-carbohydrate–low-FODMAP diet consumed during the 48 h prior to exercise was more effective in reducing exercise-associated gastrointestinal symptoms [125]. Notably, the high-carbohydrate–low-FODMAP group in this study consumed a relatively high amount of DF (53 ± 1 g/day). Therefore, high DF intake does not necessarily induce gastrointestinal symptoms during exercise. Furthermore, moderately reducing dietary fermentable oligosaccharides, disaccharides, monosaccharides, and polyols while avoiding rapidly fermented DF and selecting DF with slower and more moderate fermentation profiles may represent a more effective strategy for alleviating gastrointestinal symptoms in athletes. Rapidly fermented DF generally contains abundant β-glycosidic bonds, particularly β-2,1 linkages, which are resistant to hydrolysis by human digestive enzymes and are subsequently fermented by GM, resulting in gas production [126,127]. Therefore, avoiding rapidly fermented DF may help reduce bloating and intestinal gas while preserving gut microbial ecological balance [128].

However, not all β-glycosidic bonds in carbohydrates are associated with significant gas production. Certain types of DF, such as partially hydrolyzed guar gum (PHGG) and psyllium hull, exhibit relatively mild fermentation properties in practical applications and have good tolerance in the gastrointestinal tract [129,130]. This is because fermentability is determined not only by the type of glycosidic bond but also by other structural features, such as the degree of branching and the three-dimensional conformation, which are also crucial determinants [131]. For instance, PHGG is composed of a main chain of mannose linked by β-(1-4) glycosidic bonds and a side chain of galactose linked by α-(1-6) bonds. Its high molecular weight and branched structure may restrict the contact of microbial enzymes, slow down the degradation process, and thus result in a more gradual gas production pattern [130].

Furthermore, sex-specific considerations should be taken into account when implementing high-DF diets in athletes. This issue is particularly relevant for female athletes with inherently low BF%, such as gymnasts and endurance athletes, who may further reduce dietary energy density due to pressures related to maintaining a lean physique. A previous study investigated the effects of a low-energy-density, high-DF diet in female endurance athletes with regular menstrual cycles and those with functional hypothalamic amenorrhea (FHA) [132]. The results indicated that, although FHA athletes adopting a low-energy-density, high-DF diet tended to exhibit a leaner body phenotype, this dietary pattern was also associated with an increased risk of sports-related injuries. A high-DF diet may reduce energy intake in female athletes by lowering dietary energy density and enhancing satiety, while high-intensity exercise may further contribute to reduced energy availability [133]. Several authoritative sports medicine organizations have indicated that reduced energy availability, particularly in the context of exercise-induced physiological stress, is associated with an increased risk of the female athlete triad, which encompasses insufficient energy availability, menstrual dysfunction, and impaired bone health [134,135,136]. Therefore, when implementing high-fiber dietary strategies in female athletes, particular attention should be given to ensuring adequate energy intake, maintaining endocrine homeostasis, and conducting individualized risk assessments.

## 6. Evidence Primarily Derived from Non-Athletes: Insights into Potential Mechanisms Underlying Dietary Fiber-Mediated Regulation of Body Weight and Lipid Metabolism in Athletes

**Figure 3:**
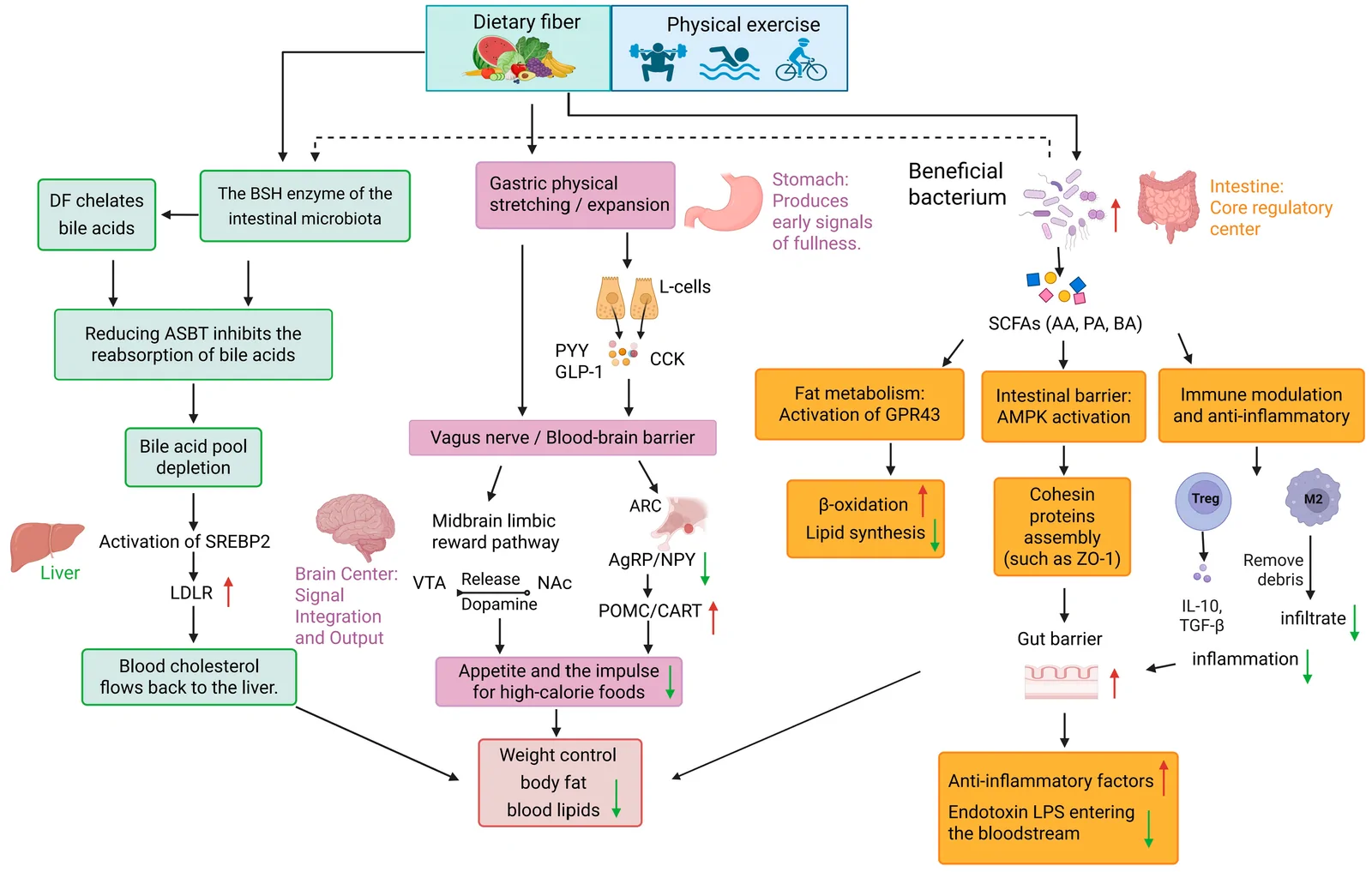
A schematic diagram illustrating how DF regulates the weight of athletes through multiple mechanisms. Dietary fiber can regulate the weight of athletes through multiple mechanisms: acting on the central nervous system to control appetite, improving lipid metabolism, and optimizing the GM and metabolites to enhance intestinal barrier and anti-inflammatory functions. DF, Dietary Fiber; BSH, Bile Salt Hydrolase; ASBT, Apical Sodium-dependent Bile Acid Transporter; SREBP2, Sterol Regulatory Element-Binding Protein 2; LDLR, Low-Density Lipoprotein Receptor; VTA, Ventral Tegmental Area; POMC, Pro-Opiomelanocortin; CART, Cocaine- and Amphetamine-Regulated Transcript; AgRP, Agouti-Related Protein; NPY, Neuropeptide Y; NAC, Nucleus Accumbens; SCFAs, Short-Chain Fatty Acids; AA, Acetic Acid; PA, Propionic Acid; BA, Butyric Acid; PYY, Peptide YY; GLP-1, Glucagon-like peptide-1; CCK, Cholecystokinin; AMPK, AMP-Activated Protein Kinase; ZO-1, Zonula Occludens-1; LPS, Lipopolysaccharide; Treg, Regulatory T Cell; M2, M2 Macrophage; IL-10, Interleukin-10; TGF-β, Transforming Growth Factor-beta; L-cells, Enteroendocrine L-cells; ARC, Arcuate Nucleus. Red arrow: upward regulatory effect; green arrow: downward regulatory effect.

DF modulates BW and lipid metabolism in athletes via multiple physiological mechanisms. These include modulation of the GM and its metabolites to strengthen intestinal barrier function and elicit anti-inflammatory effects, regulation of appetite via central nervous system pathways, and facilitation of cholesterol excretion, which collectively improve lipid profiles (Figure 3).

### 6.1. Regulating the Gut Microbiota and Associated Metabolites

The GM refers to the entire group of microorganisms (bacteria, fungi, archaea, etc.) that colonize the human digestive tract. Among them, the Firmicutes phylum and Bacteroidetes phylum are the dominant bacterial groups that can ferment DF to produce metabolites mainly consisting of SCFAs. These metabolites have been implicated in the regulation of host energy metabolism, carbohydrate and lipid metabolism, and immune responses, suggesting a potential role of the GM in BW regulation.

Current evidence suggests that DF may contribute to reductions in FM among athletes, which may be partly associated with its modulatory effects on the GM. For example, a study involving an 8-week DF supplementation intervention in basketball players reported a significant increase in the abundance of beneficial bacteria, including *Faecalibacterium prausnitzii* and *Lachnospira eligens*, within the gastrointestinal tract, which was accompanied by a concurrent reduction in FM [13]. These bacterial groups obtain energy by degrading DF and are simultaneously associated with the production of SCFAs [137,138,139,140,141]. Experimental studies in animal models suggest that SCFAs may regulate adipose tissue metabolism through GPR43 signaling in adipocytes, thereby potentially contributing to the prevention of excessive adiposity [142,143]. For instance, in obese mice supplemented with SCFAs, adipose tissue exhibited upregulation of *GPR43* gene expression and a concomitant reduction in *CPT1* mRNA levels [143]. Indeed, increased GPR43 expression has been shown to downregulate the activity of ACC, a key enzyme in de novo lipogenesis, thereby curtailing fatty acid synthesis [142,144]. Concurrently, it alleviates the inhibitory effect of malonyl-CoA on CPT1-mediated fatty acid β-oxidation [145,146]. This dual regulation thus fosters a metabolic state characterised by suppressed lipogenesis and enhanced fatty acid oxidation. However, considering the highly adaptive nature of lipid metabolism in athletes, whether DF-induced alterations in the gut microbial ecosystem contribute to FM regulation through these mechanisms remains to be elucidated.

Furthermore, DF has been shown to exert anti-inflammatory effects through modulation of the GM. In athletic populations, high-intensity exercise can compromise the stability of the intestinal barrier, primarily as a consequence of intestinal epithelial ischaemia and increased permeability [147]. The intestinal barrier, which consists of the mucus layer, epithelial cells and their intercellular tight junctions, plays a pivotal role in maintaining mucosal homeostasis. A robust barrier effectively attenuates the activation of the NF-κB-mediated pro-inflammatory signalling cascade—a process that would otherwise be triggered by the binding of deleterious agents such as LPS to Toll-like receptors. In turn, this limits the systemic burden of inflammatory cytokines, including IL6 and TNF-α, thereby mitigating their damaging effects on the host [148]. However, an increased abundance of *Akkermansia muciniphila*, *Prevotella* and *Bifidobacterium* has been observed in many athletes consuming DF [149,150,151]. Evidence from cellular studies suggests that SCFAs produced by these bacteria may activate the AMP-activated protein kinase (AMPK) signalling pathway, thereby promoting the assembly of tight junction proteins—including occludin, claudin-1 and ZO-1—and reducing paracellular permeability across the intestinal epithelium. This may subsequently limit the translocation of LPS and other inflammatory triggers into the bloodstream [152,153].

Furthermore, evidence from animal and cellular studies suggests that SCFAs, particularly butyrate, may inhibit histone deacetylase (HDAC) activity in immune cells, thereby promoting *Foxp3* gene expression. This, in turn, promotes the differentiation of naive CD4^+^ T cells into functional regulatory T (Treg) cells, which constitutively secrete anti-inflammatory cytokines, including IL-10 and TGF-β, and thus curtail mucosal pro-inflammatory responses [154,155]. Furthermore, butyrate remodels the transcriptional profile of macrophages via GPR-mediated signalling and epigenetic regulation, thereby suppressing the pro-inflammatory M1 phenotype while driving polarisation towards the anti-inflammatory M2 state. M2-polarised macrophages, in turn, employ scavenger receptors to clear apoptotic epithelial cells, endotoxins and other damage-associated debris, and concurrently downregulate chemokine expression, thereby limiting local inflammatory cell infiltration [156]. These effects may alleviate low-grade intestinal inflammation, reduce cytokine-mediated disruption of tight junctions, and synergistically preserve intestinal barrier integrity. Collectively, the enrichment of specific taxa induced by DF intake may be associated with improved intestinal barrier integrity, which could contribute to a favourable metabolic environment for BW regulation and lipid metabolism in athletes. However, most of the current evidence is derived from animal studies, and whether the enrichment of specific microbial taxa in athletes exerts these effects through similar mechanisms remains to be elucidated.

### 6.2. Suppressing Appetite

Current studies in physically active individuals and exercise model mice suggest that DF intake may help athletes regulate appetite [157,158,159]. Appetite is defined as the intrinsic physiological drive, orchestrated by the central nervous system, that impels an organism to actively seek out and ingest food. The arcuate nucleus (ARC) of the hypothalamus constitutes the principal neural centre for appetite regulation, receiving afferent projections from the vagus nerve and the nucleus tractus solitarii of the brainstem [160]. Within this nucleus, orexigenic neurons expressing AgRP/NPY and anorexigenic neurons expressing POMC/CART represent key neuronal populations involved in appetite regulation [161]. Furthermore, the mesolimbic reward pathway, comprising the ventral tegmental area (VTA) and the nucleus accumbens (NAc), also exerts an influence on appetite. This pathway receives “hunger” signals from the vagus nerve and from projections arising from the nucleus tractus solitarii of the brainstem [162,163]. In turn, it promotes the release of dopamine from VTA dopamine neurons into the NAc. When this pathway is inhibited, the urge to consume highly palatable, energy-dense foods—mediated by brain regions such as the prefrontal cortex, orbitofrontal cortex, and amygdala—is attenuated [164].

DF has been shown to influence appetite regulation through multiple physiological pathways, among which SCFAs produced by gut microbial fermentation represent an important potential mechanism. These SCFAs may modulate the secretion of gastrointestinal hormones, including glucagon-like peptide-1 (GLP-1), peptide YY (PYY), and cholecystokinin (CCK), while also suppressing ghrelin secretion [165]. These hormones modulate the activity of AgRP/NPY and POMC/CART neurons in the hypothalamus—either via vagal afferent pathways or through direct permeation of the blood–brain barrier via the systemic circulation—thereby suppressing appetite [166]. Furthermore, DF may influence appetite regulation by activating gut–brain signaling pathways through its water-holding and swelling properties. For example, in a mouse study, the swelling properties of pectin were shown to promote GLP-1 secretion through mechanical distension of the gastric wall and activate GLP-1 receptor-expressing vagal afferent neurons, which subsequently transmit satiety signals to the hypothalamus and contribute to reduced appetite [167,168]. Results showed that GLP-1 levels rose sharply within 1–2 h of intragastric pectin administration in mice, concomitant with a significant suppression of feeding behaviour. Concurrently, a marked increase in pERK1/2 (phosphorylated extracellular signal-regulated kinase) immunofluorescence was observed in the nodose ganglion (NG) and the nucleus tractus solitarii (NTS)—a highly sensitive marker of immediate neuronal activation [169]. Notably, when the vagus nerve was pretreated with capsaicin, the pectin-induced pERK1/2 activation in both the NG and NTS was almost completely abolished.

Beyond homeostatic appetite regulation, gastrointestinal hormones have also been suggested to influence hedonic feeding behaviour through modulation of the mesolimbic reward pathway. For instance, a study involving exogenous administration of PYY and GLP-1 demonstrated that, when viewing food images, participants in both the PYY and GLP-1 groups exhibited significantly reduced blood oxygenation level-dependent (BOLD) signals in the NAc and orbitofrontal cortex (OFC)—brain regions implicated in food reward-related behaviour [170]. However, a separate study on inulin supplementation yielded conflicting results. Although participants who consumed inulin for 14 consecutive days showed significantly decreased BOLD signals in the VTA and right orbitofrontal cortex (rOFC) when viewing images of highly palatable foods that they self-reported as desirable [171], correlation analyses revealed a significant positive correlation between the reduction in neuronal activity in these regions and a concomitant decline in circulating PYY levels. This discrepancy may be attributable to the fact that the measured values were fasting baseline levels rather than postprandial dynamic peak concentrations; the true appetite-regulating effects may instead manifest in response to post-meal hormonal fluctuations. Consequently, further studies employing more accurate measurement indices are warranted to validate the suppressive effects of DF on appetite and to elucidate the underlying mechanisms.

### 6.3. Promoting the Efflux of Cholesterol

Cholesterol homeostasis plays a critical role in maintaining human health, particularly in regulating lipid metabolism and cardiovascular health. Cholesterol can bind to apolipoproteins to form different lipoprotein particles, including very-low-density lipoprotein cholesterol (VLDL-C), LDL-C, and HDL-C [172]. Elevated circulating levels of VLDL-C and LDL-C are known to promote vascular obstruction and play a pivotal role in the pathogenesis of atherosclerotic cardiovascular disease (ASCVD) [173]. Although athletes are generally considered to exhibit more favourable lipid profiles due to their high levels of habitual physical activity and regular exercise training [20], accumulating evidence indicates that dyslipidaemia remains relatively prevalent among athletes [174]. This phenomenon may be partly associated with athletes’ dietary patterns, such as the long-term adoption of high-fat, low-carbohydrate diets, which have been reported to increase circulating cholesterol concentrations [175]. Therefore, maintaining cholesterol metabolic homeostasis represents an important consideration for athlete health. Current evidence suggests that DF facilitates cholesterol elimination by enhancing the excretion of cholesterol and bile acids [176,177].

DF has been demonstrated to promote cholesterol efflux. Under normal physiological conditions, cholesterol is converted into bile acids via the catalytic action of cholesterol 7α-hydroxylase (CYP7A1), after which these bile acids are secreted into the intestinal lumen [178]. Upon reabsorption in the ileum, they activate the nuclear FXR within ileal enterocytes, leading to the induction of fibroblast growth factor 19 (FGF19) [179]. Upon entering the bloodstream via the portal vein and reaching the liver, FGF19 binds to the FGFR4/β-Klotho receptor complex on the surface of hepatocytes, thereby suppressing transcription of the *CYP7A1* gene [178]. This negative feedback mechanism curtails de novo bile acid synthesis and consequently helps maintain the stability of the circulating bile acid pool. However, DF can sequester bile acids via hydrogen bonding and hydrophobic interactions. This sequestration reduces the reabsorption of bile acids by the apical sodium-dependent bile acid transporter (ASBT) in the ileum, thereby promoting de novo bile acid synthesis [180]. This process not only reduces intestinal fat absorption but also indirectly promotes cholesterol efflux. In addition, several studies have indicated that DF enriches gut microbial populations possessing bile salt hydrolase (BSH) activity—including *Bifidobacterium*, *Lactobacillus* and *Akkermansia muciniphila*—which catalyse the deconjugation of bile acids to yield free primary bile acids, such as cholic acid and deoxycholic acid [181,182]. Alternatively, these free bile acids may undergo further 7α-dehydroxylation to generate secondary bile acids, including deoxycholic acid and lithocholic acid [182]. This modification further diminishes the recognition of bile acids by the ASBT in the ileum, thereby reducing their reabsorption.

It should be noted that many contemporary cholesterol studies rely on murine models. However, the rates of cholesterol and bile acid turnover in humans are generally slower than those in mice. This difference arises because rodents exhibit higher basal rates of cholesterol synthesis and bile flow and rely predominantly on HDL—rather than LDL-C—for systemic cholesterol transport [183,184]. However, LDL is the main cholesterol carrier in the circulation [185]. Consequently, cholesterol homeostasis in humans is more critically dependent on hepatic LDL receptor (LDLR)-mediated clearance of LDL than in mice. Notably, when intrahepatic cholesterol levels decline—such as during enhanced bile acid synthesis—the sterol regulatory element-binding protein 2 pathway is activated, leading to transcriptional upregulation of LDLR expression [186]. The LDLR binds circulating LDL-C and mediates its endocytosis into hepatocytes, where the internalized cholesterol is released and utilized for cellular needs or re-esterification. This feedback mechanism helps maintain homeostasis of the gut–liver cholesterol pool and ultimately contributes to improved systemic lipid profiles.

However, it should be noted that lipid profiles may vary substantially across different sports disciplines. For example, a large-scale study involving Olympic athletes demonstrated that endurance athletes exhibited the most favourable lipid profile, characterized by the highest HDL-C concentrations and the lowest TG levels [187]. In contrast, skill-based athletes displayed the lowest HDL-C levels and a higher prevalence of lipid abnormalities. These differences may be partly attributed to the distinct metabolic adaptations induced by different types of exercise. Aerobic exercise improves lipid metabolism primarily through enhanced LPL activity and increased fatty acid metabolism, whereas resistance training supports long-term lipid control mainly by improving insulin sensitivity and reducing hepatic triglyceride production [188]. In addition, female athletes generally exhibit more favourable lipid metabolism profiles, which may be partly explained by the beneficial effects of higher oestrogen levels on lipid regulation [95,189]. Therefore, when applying DF interventions to promote cholesterol homeostasis in athletes, sport type, training characteristics, and sex-specific physiological factors should be considered to enable individualized assessment and nutritional strategies.

## 7. Research Limitations and Future Directions

Although some studies have demonstrated the benefits of DF and high-DF diets for BW regulation in athletes, most have failed to comprehensively capture the distinct phases of an athlete’s training and competitive cycle—including daily living, training, and competition. Moreover, existing longitudinal investigations have predominantly focused on continuous or single-bout endurance activities (e.g., long-distance running) or weight-category sports, whereas research on ball- and team-based sports—which typically involve high-intensity intermittent exercise—remains largely cross-sectional. Future research should therefore prioritize long-term, longitudinal designs across a broader spectrum of sports disciplines. In addition, reliance solely on self-reported dietary records should be avoided to mitigate inherent recall bias and measurement error [190].

In addition, traditional sports nutrition guidelines have often recommended reducing or avoiding DF intake during competition to mitigate the risk of gastrointestinal symptoms [191]. However, not all DF types elicit this adverse response. For instance, in a marathon study, capsules containing sodium alginate and fructose-encapsulated ultra-high-dose carbohydrates enabled a more stable race pace compared with conventional carbohydrate capsules [192]. Upon entering the acidic gastric environment, sodium alginate and pectin rapidly form a microscopic “hydrogel matrix” via acid-induced gelation, thereby facilitating accelerated gastric emptying. When this hydrogel reaches the mildly alkaline milieu of the small intestine, it spontaneously and rapidly dissociates, releasing a high concentration of sugars for efficient absorption. These findings suggest that the strategic selection and application of DF—tailored to its physicochemical properties—can positively influence athletic performance. Looking ahead, DF and related polysaccharides may be conceptualized as dynamic, programmable “physiological switches” that play integral roles in metabolic and immune regulation.

Furthermore, athletes across different sports disciplines display marked differences in energy utilization patterns, energy requirements, and body composition goals [193]. Even within the same sport, substantial inter-individual variability persists in baseline GM composition, microbial fermentation capacity, and gastrointestinal (GI) tolerance [194]. Consequently, future research should prioritize rigorously designed longitudinal studies and randomized controlled trials to systematically elucidate the long-term effects and underlying causal mechanisms of DF interventions across diverse athletic contexts. Standardized characterization of DF interventions—including DF type, physicochemical properties, fermentation kinetics, and dosage—is critical to enhance cross-study comparability and reproducibility. Furthermore, future research should incorporate comprehensive assessments of energy supply, gastrointestinal tolerance, and exercise-induced gastrointestinal symptoms to inform microbiome-informed [195] personalized dietary fiber recommendations.

## 8. Conclusions

DF and high-DF diets offer a promising novel strategy for regulating BW and lipid metabolism in athletes. However, while the physiological mechanisms through which DF exerts its effects are well-established in general populations and basic research, the direct evidence for these cascading effects specifically in athletes remains preliminary and warrants further validation. Based on these established mechanisms, DF is hypothesized to regulate appetite via gut–brain signaling involving vagal afferents, satiety hormones such as GLP-1 and PYY, and SCFA-mediated central pathways. Similarly, the lipid-lowering benefits are thought to be mediated through enhanced cholesterol excretion via the enterohepatic bile acid circulation, suppression of de novo lipogenesis, promoting reverse cholesterol transport, and stimulating AMPK-driven fatty acid oxidation. Furthermore, by enriching beneficial GM (e.g., *Akkermansia* and *Prevotella*) and reinforcing intestinal barrier integrity, DF may help alleviate chronic inflammation. Nevertheless, it is important to emphasize that the direct translation and universal manifestation of these pathways in athletes require further confirmation through sport-specific intervention trials. Despite these promising benefits, the acceptance of DF among athletes remains low, largely due to the risk of provoking gastrointestinal discomfort during training and competition. The type of glycosidic bonds, the degree of side-chain branching, and the three-dimensional conformation of DF are key determinants of its degradation behavior, which can be optimized by rationally selecting DF sources and intake timing. Future research in this area should prioritize well-controlled, long-term longitudinal studies and randomized controlled interventions with standardized characterization of fiber types and dosages. Critically, these future trials must carefully assess energy availability and gastrointestinal symptoms in parallel, given the unique physiological demands and susceptibility of athletes. Building upon this robust foundational evidence, future efforts can then safely explore precision-nutrition approaches tailored to individual metabolic phenotypes and microbiome profiles, aiming to optimize both health outcomes and athletic performance.

## Data Availability

No new data were created or analyzed in this study. Data sharing is not applicable to this article.

## Abbreviations

The following abbreviations are used in this manuscript:

DF	Dietary fiber
BW	Body weight
high-DF diets	High-dietary-fiber diets
GM	Gut microbiota
SCFAs	Short-chain fatty acids
AMPK	AMP-activated protein kinase
TG	Triglyceride
BF%	Body-fat percentage
LDL-C	Low-density lipoprotein cholesterol
TC	Total cholesterol
HDL-C	High-density lipoprotein cholesterol
BAT	Brown adipose tissue
SNS	Sympathetic nervous system
cAMP	Cyclic adenosine monophosphate
PKA	Protein kinase A
UCP-1	Uncoupling protein 1
WAT	White adipose tissue
HDL	High-density lipoprotein
LBM	Lean body mass
LPS	Lipopolysaccharide
GLP-1	glucagon-like peptide-1
FXR	Farnesoid X receptor
VLDL	Very-low-density lipoprotein
SDF	Soluble dietary fiber
IDF	Insoluble dietary fiber
FM	Fat mass
VO_2_ max	Maximal oxygen uptake
VD	Vegetarian diet
MD	Mediterranean diet
EPA	Eicosapentaenoic acid
DHA	Docosahexaenoic acid
CPT-1	Carnitine palmitoyltransferase 1
ALAT	Alanine aminotransferase
PRAL	Potential renal acid load
NEAP	Net endogenous acid production
PHGG	Partially hydrolyzed guar gum
FHA	Functional hypothalamic amenorrhea
WFPB	Whole food plant-based
PBMA	Plant-based meat alternatives
FFM	Fat-free mass
SMM	Skeletal muscle mass
BCMI	Body cell mass index
TBW	Total body water
BMR	Basal Metabolic Rate
KIDMED	Mediterranean diet quality index for children and adolescents
WC	Waist circumference
HDAC	Histone deacetylase
ARC	Arcuate nucleus
AgRP/NPY	Agouti-related protein/neuropeptide Y
POMC/CART	Pro-opiomelanocortin/cocaine- and amphetamine-regulated transcript
VTA	Ventral tegmental area
PYY	Peptide YY
CCK	Cholecystokinin
NG	Nodose ganglion
NTS	Nucleus tractus solitarii
BOLD	Blood oxygenation level-dependent
OFC	Orbitofrontal cortex
CYP7A1	Cholesterol 7α-hydroxylase
FGF19	Fibroblast growth factor 19
